# A Constitutive Model for Describing the Tensile Response of Woven Polyethylene Terephthalate Geogrids after Damage

**DOI:** 10.3390/ma16155384

**Published:** 2023-07-31

**Authors:** Giovani Lombardi, Margarida Pinho-Lopes, António Miguel Paula, António Bastos Pereira

**Affiliations:** 1 RISCO, Department of Civil Engineering, University of Aveiro, Campus Universitário de Santiago, 3810-193 Aveiro, Portugal; mlopes@ua.pt (M.P.-L.); mpaula@ipb.pt (A.M.P.); 2 CONSTRUCT-GEO, Department of Civil Engineering, Faculty of Engineering, University of Porto, Rua Doutor Roberto Frias, s/n, 4200-465 Porto, Portugal; 3 Instituto Politécnico de Bragança, Campus de Santa Apolónia, 5300-253 Bragança, Portugal; 4 TEMA, Department of Mechanical Engineering, University of Aveiro, Campus Universitário de Santiago, 3810-193 Aveiro, Portugal; 5 LASI—Intelligent Systems Associate Laboratory, 4800-058 Guimarães, Portugal

**Keywords:** geosynthetics, constitutive models, damage, statistical analysis

## Abstract

A constitutive model was used to describe the tensile response of two woven Polyethylene Terephthalate (PET) geogrids, before and after mechanical damage. The model parameters of undamaged and damaged specimens were estimated via numerical regressions of test results. For each sample, the experimental and fitted tensile strengths were statistically compared using hypothesis tests. For each geogrid, tensile load–strain curves of damaged samples were drawn by applying scaling factors to the plot of the undamaged sample. The curve fittings resulted in high R^2^ values for undamaged and damaged specimens of the geogrids. For most samples, there was no significant mean difference between the experimental and fitted tensile strength. The model allowed us to describe the load–strain curve of a geogrid from its tensile properties: ε*_max_*, *T_max_* and *J_i_*. Regardless of the type of damage (in laboratory or in situ), the model was able to describe the load–strain curves of damaged samples using data from undamaged samples and scaling factors.

## 1. Introduction

Geosynthetic is a generic name given to planar products, mostly composed of polymers, and used in contact with soil, rock or with any other material as part of a constructive system [1]. Most geosynthetics are composed of thermoplastic polymers, such as polypropylene, polyester, polyethylene, polyvinyl, polyamide, and polystyrene [2].

Geosynthetics have been increasingly applied in civil engineering and geotechnical works due to the advantages presented when compared to traditional solutions. Geosynthetics have several important functions and can be applied in a wide range of structures, namely, reinforced soils, support walls, very steep slopes, landfills for waste disposal, erosion control and coastal protection [2]. Geosynthetics can contribute to a more resilient and sustainable world, as they may provide quality water, protect the environment, mitigate natural disasters, use more economical solutions and connect people [3].

Among the different applications of geosynthetics, their use in roads can be highlighted. Geosynthetics are used in both paved and unpaved roads [4] to perform different functions [5]: reinforcement, stabilisation, stress-relief interlayer, separation, fluid barrier, drainage, and filtration. Roads (paved or unpaved) can be improved via mechanical means, and the relevant functions are reinforcement and stabilisation [5]; thus, the mechanical response of geosynthetics is key for the design. Similarly, there are other projects where geosynthetics act as reinforcements so that the short- and long-term mechanical responses are primary aspects in the design, such as in retaining walls and reinforced soil slopes [6,7,8]. Models that can realistically represent the tensile response of geosynthetics are fundamental for attaining economic solutions. In the literature, there are examples of studies using a variety of constitutive models for geosynthetics [9,10,11,12].

Different constitutive theories can form the basis of the stress–strain relation of materials, such as elasticity, plasticity, viscoelasticity and viscoplasticity [13]. The mechanical behaviour of geosynthetics is a combination of the typical response of an elastic solid, a viscous liquid, and a plastic, depending primarily on the temperature [14].

The tensile response of geosynthetics is affected by several factors, including the type and arrangement of the constituent polymer, environmental conditions, soil confinement, and loading level, rate and duration [15]. Physical models (e.g., rheological and damage models) and mathematical models (e.g., polynomial and hyperbolic models) are used to describe the tensile load–strain response of geosynthetics. Physical models are employed to account for microscopic effects, whereas mathematical models are phenomenological—they only represent experimental results and do not consider microscopic effects [16].

Hyperbolic equations to model the behaviour of geosynthetics and reinforced soil structures were reported by [17,18,19,20,21,22,23,24]. Bathurst and Naftchali [25] stated that these equations are significantly accurate for analytical and numerical modelling of geosynthetics; the authors reported that the model parameters were related to the strain rate and the curvature of the tensile load–strain plot. Ezzein et al. [26] also supported that the parameters of hyperbolic models depend on the strain rate.

**Figure 1:**
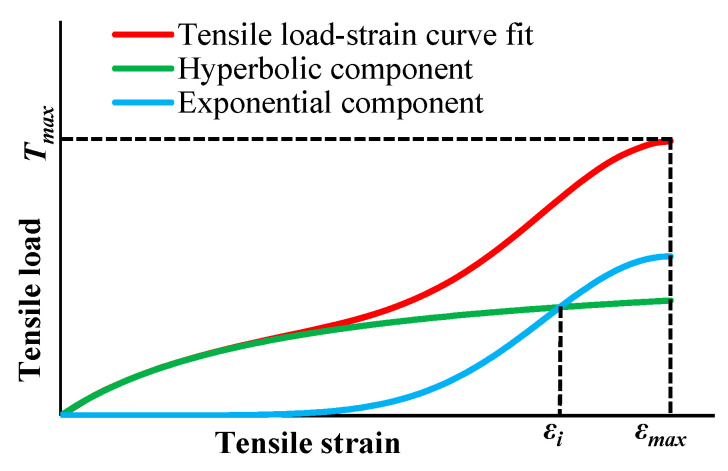
Typical tensile load–strain curve of a geosynthetic that presents a stiffening response. Components of the constitutive model (Equation (1)).

In order to capture the stiffening in the tensile load–strain response of some geosynthetics, the nonlinear model given in Equation (1) combines an exponential function to fit high strains and a hyperbola to fit low strains (Figure 1). The tangent stiffness (Equation (2)) is given by the derivative of Equation (1) with respect to the tensile strain [19].

(1) $$T=\frac{\varepsilon }{\left(a+2\cdot b\cdot \varepsilon \right)}+\frac{1}{2\cdot b}\cdot e^{-c\cdot (\varepsilon \;-\varepsilon_{\max}{)}^{2}}\;(\varepsilon \;\geq \;0)$$

(2) $$J=\frac{\mathrm{dT}}{d\varepsilon }=\frac{a}{{\left(a+2\cdot b\cdot \varepsilon \right)}^{2}}-\frac{c\cdot \left(\varepsilon \;-\varepsilon_{\max}\right)}{2\cdot b}\cdot e^{-c\cdot {\left(\varepsilon \;-\varepsilon_{\max}\right)}^{2}}\;(\varepsilon \;\geq \;0)$$

*T*: tensile load per unit width;
*J*: tangent stiffness;
*ε*: tensile strain;
*a*, *b*, and *c*: model parameters;
*ε_max_*: strain at maximum load.

Geosynthetics are subjected to damage mechanisms in storage, during construction and post-installation, including weathering, chemicals, high temperatures, abrasion, creep, and oxidation. The durability of a geosynthetic depends on several factors, such as atmospheric agents, the type of polymer, its structure, and its primary function [15].

The assessment of durability is based on experimental observations and tests performed to evaluate damages expected during the design life of a geosynthetic. The lifespan of a geosynthetic is usually estimated based on gradual changes in physical and mechanical properties, increasing deformations, reductions in strength and stiffness, holes, or any other change that might compromise its performance and durability [27].

In particular, damage occurring during installation (DDI) may modify the structure of geosynthetics. Noticeable cuts and holes, detachment and disaggregation of the coating surface are typical consequences due to placement, spreading and compaction of backfill material over a geosynthetic. DDI is immediate, resulting in a rapid and irreversible reduction in stiffness and strength, thus being part of the durability assessment [27].

**Figure 2:**
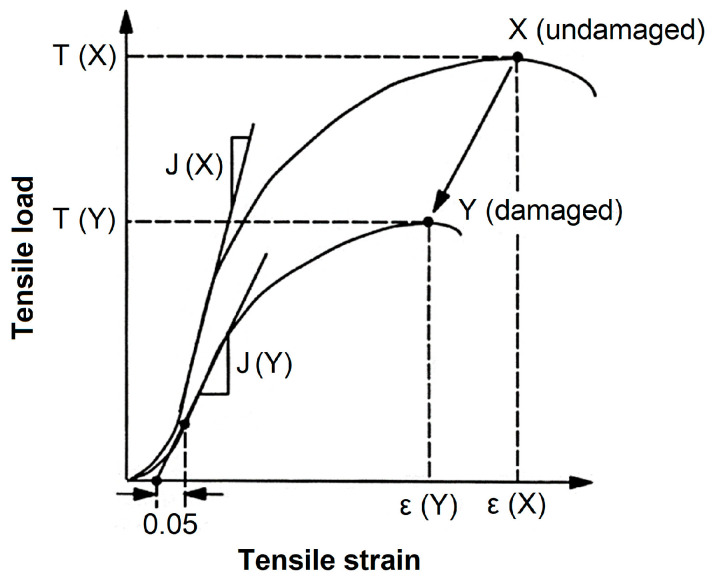
Changes in the short-term tensile response of a geosynthetic after damage [28]—adapted.

Bathurst and Allen [28] reported that the short-term tensile load–strain curve of a damaged geosynthetic can be described by applying scaling factors to the plot of an undamaged sample (Figure 2). In this sense, the following three scaling factors are used to characterize the tensile response after damage: peak strength retained: *R_T_* (Equation (3)); modulus retained: *R_J_* (Equation (4)); and peak strain retained: *R_ε_* (Equation (5)). The authors concluded that *R_T_*, *R_J_* and *R_ε_* can be used if the shape of the load–strain curve is not significantly modified after damage, and variability in the data and measurements are considered.

(3) $$R_{T}=\frac{T_{\max}(Y)}{T_{\max}(X)}$$

(4) $$R_{J}=\frac{J_{i}\left(Y\right)}{J_{i}\left(X\right)}$$

(5) $$R_{\varepsilon }=\frac{\varepsilon_{\max}\left(Y\right)}{\varepsilon_{\max}\left(X\right)}$$

*R_T_*, *R_J_* and *R_ε_*: scaling factors;
*X*: undamaged sample;
*Y*: damaged sample.

The objectives of this study are summarized as follows.

- Apply a constitutive model to describe the short-term tensile response of undamaged and damaged specimens of two woven Polyethylene Terephthalate (PET) geogrids; estimate the model parameters; assess the goodness of the fits; statistically compare experimental and fitted data.
- Determine the scaling factors by relating the tensile properties of undamaged and damaged samples of the geogrids.
- For each geogrid, obtain the tensile load–strain curve of damaged samples by applying scaling factors to the plot of the undamaged sample; assess the goodness of the fits; statistically compare predicted and fitted data.

## 2. Materials

Data from two woven PET geogrids (Table 1) were analysed. Specimens of both geogrids were damaged in a laboratory (MEC) following EN ISO 10722 [29], in which the specimens are placed between layers of a synthetic aggregate, and then submitted to cyclic loading ranging between 5 kPa and 500 kPa, at a frequency of 1 Hz for 200 cycles. The experimental data and the procedures of damage were reported by [23,30,31].

Specimens of the geogrid GWP60 were submitted to damage during installation: they were placed between layers of granite residual soil, and then they were subjected to two distinct levels of compaction energy as per the Proctor’s test: 90% (DDI90) and 98% (DDI98). The test beds were constructed on a road-building site, over a road platform. The geosynthetics were placed on top of a 0.20 m soil lift, properly spread, levelled, and compacted. Two additional soil lifts were placed over the geosynthetics, each 0.20 m high, for a total height of 0.60 m. A vibratory roller was utilized to compress the soil. The experimental data and the procedures of damage in situ were reported by [32].

Thereafter, undamaged (UND) and damaged specimens of the geogrids were subjected to tensile tests following EN ISO 10319 [33], where the strains were measured by video extensometers at short intervals (about 0.3 s). The following tensile properties were determined from the test results: the tensile strength (*T_max_*) and the strain at *T_max_* (*ε_max_*).

## 3. Methods

Although recent studies have shown a relationship between the parameters of hyperbolic models and the strain rate, the results analysed here were obtained for specimens tested at a constant strain rate: 20 ± 5%/minute, as per EN ISO 10319 [33]. Thus, the effect of the strain rate was not considered in this study. In addition, this paper reports results for geosynthetics that present a stiffening response, as shown in Figure 1.

**Figure 3:**
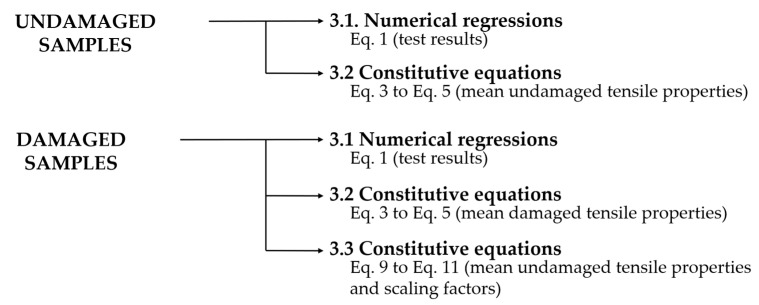
Methods used to estimate the model parameters, detailed in Section 3.1, Section 3.2 and Section 3.3.

Figure 3 illustrates the methods used to estimate the model parameters of undamaged and damaged samples, detailed in Section 3.1, Section 3.2 and Section 3.3. Table 2 summarizes the main terms and definitions addressed in the following topics.

### 3.1. Numerical Regressions (Curve Fittings)

A constitutive model (Equation (1)) was applied to describe the tensile load–strain recurves of undamaged and damaged specimens of the geogrids. The higher the sample size, the more robust the statistical analysis.

SPSS^®^ was used to fit experimental data, and the model parameters were estimated via nonlinear regressions according to the Levenberg–Marquardt algorithm. The model parameters were estimated with confidence intervals of 95%, and the coefficient of determination (R^2^ value) was used to assess the goodness of the fits.

With confidence intervals of 95%, data were statistically compared using the Student *t*-test—a hypothesis test for independent samples, applied to compare the difference in means between two samples of normally distributed data [34]. The tests of normality were performed in SPSS^®^ using the Shapiro–Wilk method—applied to small sample sizes [35]. Levene’s tests provided the homogeneity of variance.

The hypothesis tests were used to compare mean values (e.g., experimental vs. fitted *T_max_*), while the R^2^ values were used to assess the goodness of the fits (along the entire plot). For both undamaged and damaged samples of the geogrids, the mean estimates of model parameters were used to plot the representative tensile load–strain curve (mean curve). Other representative curves were assessed (Table 2), but the mean curve resulted in the highest R^2^ values for all samples analysed.

### 3.2. Mathematical Relations between the Model Parameters and the Tensile Properties

The relations between the model parameters to each other and the tensile properties are mathematically determined by applying boundary conditions to Equations (1) and (2). Equation (6) is determined from Equation (2) (*ε* → 0), and it relates the model parameter a to the initial tangent stiffness (*J_i_*); Equation (6) was reported by [19].

Equation (7) is obtained from Equation (1) (*ε* → *ε_max_*); it relates the model parameter *b* to the tensile strength (*T_max_*), the strain at maximum load (*ε_max_*) and the model parameter *a*. Lastly, Equation (8) is deduced from Equation (1) (*ε* → *ε_i_*, Figure 1), and relates the model parameter *c* to *ε_max_* and the parameters *a* and *b*.

(6) $$a=\frac{1}{J_{i}}\;(\varepsilon \;\to \;0)$$

(7) $$b=\frac{-a\cdot T_{\max}+2\cdot \varepsilon_{\max}+\sqrt{4\cdot \varepsilon_{\max}{}^{2}+a^{2}\cdot T_{\max}{}^{2}}}{4\cdot \varepsilon_{\max}\cdot T_{\max}}\;(\varepsilon \;\to \;\varepsilon_{\max})$$

(8) $$c=\frac{-\ln\left(\frac{2\cdot b\cdot \varepsilon_{i}}{a+2\cdot b\cdot \varepsilon_{i}}\right)}{{\left(\varepsilon_{i}-\varepsilon_{\max}\right)}^{2}}\;(\varepsilon \;\to \;\varepsilon_{i})\;\left\{\varepsilon_{i}\in {\mathbb{R}}^{+}/\varepsilon_{i}\neq 0,\varepsilon_{i}\neq \frac{-a}{2\cdot b},\varepsilon_{i}\neq \varepsilon_{\max}\right\}$$

*J*_*i*_: initial tangent stiffness;
*T_max_*: tensile strength;
*ε_i_*: strain for which the hyperbolic and exponential components intersect (Figure 1).

For each damaged and undamaged sample of the geogrids, the model parameters were determined from Equation (6) to Equation (8) using mean experimental *ε_max_*, and *T_max_* and *J_i_* were fitted by the representative curve. *ε_i_* was determined via iteration using the bisection method (there is a value of *ε_i_* for which the components of the constitutive model intersect, as shown in Figure 1). The model parameters determined from these equations were compared to those estimated via numerical regressions of test results.

### 3.3. Damaged Curves Described Using Undamaged Data and Scaling Factors

For each damaged sample of the geogrids, the model parameters were predicted from undamaged data using Equations (9)–(11). These equations are analogous to Equation (6) to Equation (8), where the damaged tensile properties were determined from Equation (3) to Equation (5) (using mean undamaged tensile properties and scaling factors). The values for model parameters determined from these equations were compared to those estimated via numerical regressions of test results.

(9) $$a(Y)=\frac{1}{J_{i}(Y)}$$

(10) $$b(Y)=\frac{\frac{-T_{\max}(Y)}{J_{i}(Y)}+2\cdot \varepsilon_{\max}(Y)+\sqrt{4\cdot {\left[\varepsilon_{\max}(Y)\right]}^{2}+{\left[\frac{T_{\max}(Y)}{J_{i}(Y)}\right]}^{2}\cdot {\left[T_{\max}(Y)\right]}^{2}}}{4\left[\varepsilon_{\max}(Y)\right]\cdot \left[T_{\max}(Y)\right]}$$

(11) $$c(Y)=\frac{-\ln\left(\frac{2\cdot b(Y)\cdot \varepsilon_{i}(Y)}{a(Y)+2\cdot b(Y)\cdot \varepsilon_{i}(Y)}\right)}{{\left[\varepsilon_{i}(Y)-\varepsilon_{\max}(Y)\right]}^{2}}$$

*T_max_*(*Y*), *J_i_*(*Y*) and *ε_max_*(*Y*): damaged tensile properties (from Equation (3) to Equation (5)).

## 4. Results and Discussions

**Figure 4:**
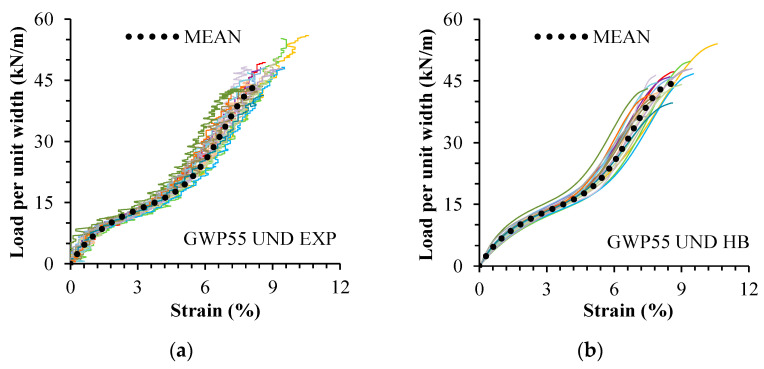

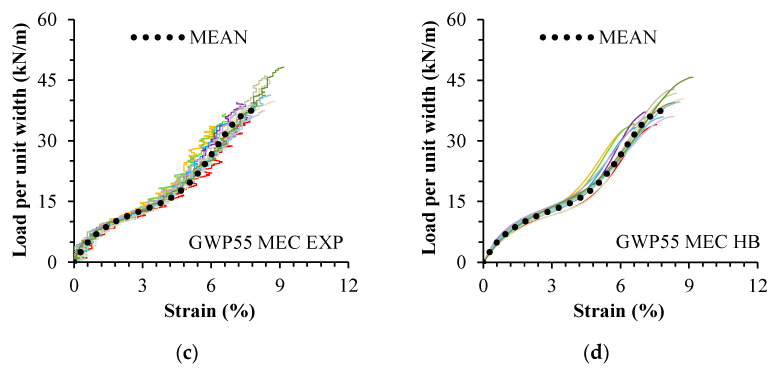
Geogrid GWP55. Experimental curves (EXP) and fitted curves (HB): (**a**) UND EXP; (**b**) UND HB; (**c**) MEC EXP; (**d**) MEC HB. Undamaged (UND). Damaged in laboratory (MEC). Each color represents a test.

**Figure 5:**
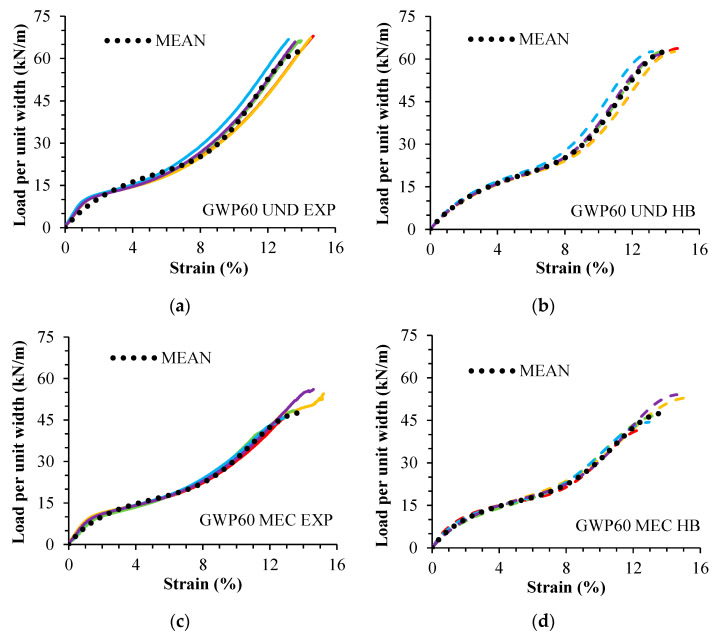

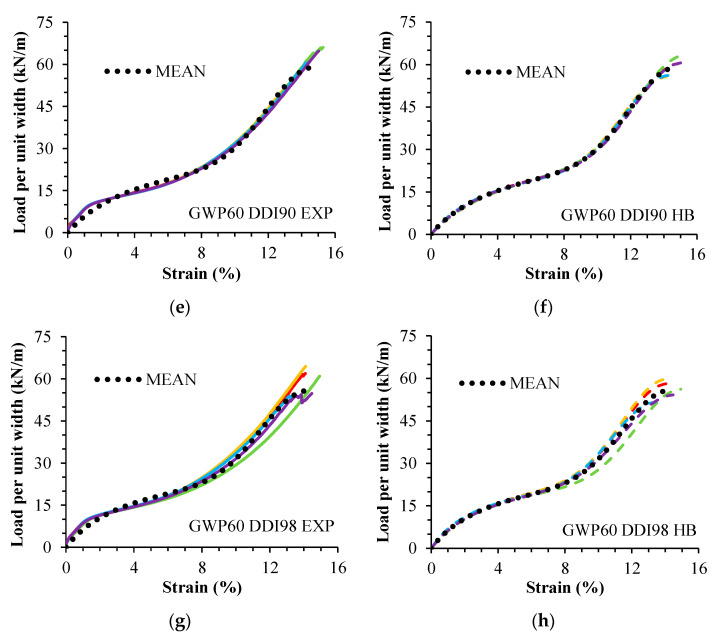
Geogrid GWP60. Experimental curves (EXP) and fitted curves (HB): (**a**) UND EXP; (**b**) UND HB; (**c**) MEC EXP; (**d**) MEC HB; (**e**) DDI90 EXP; (**f**) DDI90 HB; (**g**) DDI98 EXP; (**h**) DDI98 HB. Undamaged (UND). Damaged in laboratory (MEC). Damaged during installation (DDI). Each color represents a test.

The model (Equation (1)) was able to qualitatively describe the tensile response of undamaged and damaged specimens of both geogrids, regardless of the type of damage (in laboratory or in situ). The curve fittings resulted in high R^2^ values (between 0.979 and 0.997). Table 3 gives the mean experimental and fitted tensile properties. The experimental and fitted tensile load–strain curves are presented in Figure 4 (GWP55) and Figure 5 (GWP60).

Table 4 gives the sample sizes, the mean parameter estimates, the tensile properties fitted by the mean curve, and the scaling factors. All experimental and fitted data are normally distributed. The hypothesis tests indicated that there was no significant mean difference between the experimental and fitted tensile strength, except for GWP60 UND.

For undamaged and damaged samples of the geogrids, the values for model parameters determined from Equation (6) to Equation (8) were equal to those estimated via numerical regressions, which reinforces the mathematical relations between the model parameters to each other and the tensile properties, as proposed in these equations.

**Figure 6:**
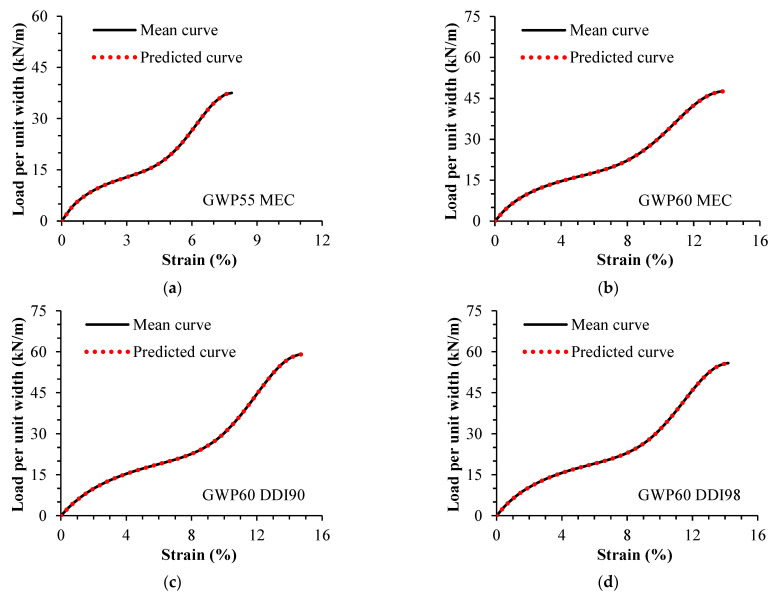
Damaged curves: (**a**) GWP55 MEC; (**b**) GWP60 MEC; (**c**) GWP60 DDI90; (**d**) GWP60 DDI98. Mean curve: plotted using mean parameter estimates. Predicted curve: plotted using the damaged parameters predicted from Equation (9) to Equation (11). Damaged in laboratory (MEC). Damaged during installation (DDI).

Table 5 gives the predicted parameters (from Equation (9) to Equation (11)), and the mean parameter estimates (from numerical regressions using Equation (1)) for damaged samples. Figure 6 shows the representative and predicted curves of damaged samples. Values for damaged parameters predicted from Equation (9) to Equation (11) were equal to those estimated via numerical regressions, which demonstrates the capacity of the model to describe damaged curves from undamaged data using scaling factors.

## 5. Conclusions

In this study, a constitutive model (Equation (1)) was applied to fit the tensile response of two woven PET geogrids, before and after damage. The model parameters were estimated via numerical regressions of experimental data. Values for the model parameters were determined from Equation (6) to Equation (8) using mean tensile properties. Values for the model parameters of damaged samples were also determined from Equation (9) to Equation (11) using undamaged data and scaling factors. For each sample, hypothesis tests were used to statistically compare the experimental and fitted tensile strength (mean values). For each specimen, the R^2^ value was used to assess the goodness of the fit. The main conclusions of the research are stated as follows.

- The model was able to qualitatively describe the tensile load–strain response of undamaged and damaged specimens of both geogrids (high R^2^ values).
- If compared to experimental values, the model proved capable of fitting the tensile strength of most samples of the geogrids (for most samples, there was no significant mean difference between the experimental and fitted tensile strength).
- The model allowed us to describe the tensile load–strain curve of a geogrid (before and after damage) only from its tensile properties: $\varepsilon_{max}$, $T_{max}$ and $J_{i}$.
- Regardless of the type of damage, the model was able to describe tensile load–strain curves of damaged samples using data from undamaged samples and scaling factors.

This paper presented a successful approach to predict the short-term tensile response of two woven geogrids after mechanical damage induced in a laboratory and after damage during installation. The estimates were based on the tensile properties of the undamaged materials and scaling factors—relating the tensile properties of the damaged samples with those of the reference material (undamaged sample). Thus, experimental data from damaged and undamaged specimens are required to determine the scaling factors.

This approach has the potential for being further extended and applied in the design of geosynthetics as the scaling factors of a geogrid could be estimated using such information—when a robust database is available for comparable conditions (geosynthetics and damage/installation conditions). Therefore, this approach could allow the prediction of the tensile load–strain curve of a damaged geogrid before test data are available.

## Figures and Tables

**Table 1:** Nominal properties of the geosynthetics.

Geosynthetic	GWP55		GWP60	
Type	Geogrid	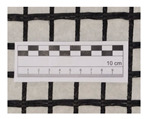	Geogrid	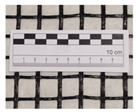
Structure	Woven		Woven	
Constituent polymer	PET		PET	
Nominal tensile strength (kN/m)	55		60	
Nominal tensile strain (%)	10.5		14.0	
Grid spacing (mm × mm)	25 × 25		20 × 20	

**Table 2:** Main terms and definitions.

Term	Symbol	Definition
Model parameters	a , b , c	Parameters of the constitutive model (Equation (1))
Parameter estimates	–	Model parameters estimated via numerical regressions of experimental data
Mean parameter estimates	–	Mean estimates of the model parameter of a sample
Median parameter estimates	–	Median estimates of the model parameter of a sample
Tensile properties	$J_{i}$ $,\;T_{max}$ $,\;\varepsilon_{max}$	Tensile properties of a certain geogrid
Mean undamaged tensile properties	–	Mean experimental tensile properties of an undamaged sample
Mean damaged tensile properties	–	Mean experimental tensile properties of a damaged sample
Predicted damaged parameters	$a\left(Y\right)$ $,\;b\left(Y\right)$ $,\;c\left(Y\right)$	Model parameters for the response after damage predicted from undamaged data using scaling factors (Equations (3)–(5))
Representative curve:	–	Load–strain curve that best represents the trends in the data of a sample
Mean curve ^$^	–	Load–strain curve plotted using mean parameter estimates
Median curve	–	Load–strain curve plotted using median parameter estimates
Intermediate curve	–	Experimental load–strain curve that visually is in an intermediate position relative to the other curves of a sample

^$^ mean parameter estimates were used to plot the representative curves.

**Table 3:** Mean experimental and fitted tensile properties.

	Mean Experimental Tensile Properties		Mean Fitted Tensile Properties (Equation (1))	
Sample	*ε_max_*	*T_max_*	*T_max_*	*J_i_*
	%	kN/m	kN/m	kN/m
GWP55 UND	8.5	46.72	44.66	957.03
GWP55 MEC	7.8	39.80	37.88	938.93
GWP60 UND	14.0	66.84	62.70 *	734.53
GWP60 MEC	13.8	50.11	48.16	744.54
GWP60 DDI S90	14.7	63.01	59.19	708.04
GWP60 DDI S98	14.2	59.23	55.99	786.16

*T_max_*: tensile strength; *ε_max_*: strain at *T_max_*; *J_i_*: initial tangent stiffness; * significant mean difference.

**Table 4:** Mean parameter estimates. Tensile properties fitted by the mean curve. Scaling factors.

	Sample	Mean Parameter Estimates			Tensile Properties		Scaling Factors		
Sample	Size	Equation (1) (SPSS^®^)			Mean Curve		Equation (3)	Equation (4)	Equation (5)
	*N*	*a*	*b*	*c*	*T_max_*	*J_i_*	$R_{T}$	$R_{J}$	$R_{\varepsilon }$
	–	m/kN	m/kN	–	kN/m	kN/m	–	–	–
GWP55 UND	20	0.1085	0.0198	0.1763	44.28	921.50	–	–	–
GWP55 MEC	15	0.0936	0.0240	0.1957	37.55	1068.35	0.848	1.159	0.917
GWP60 UND	5	0.1364	0.0139	0.0703	62.69	733.18	–	–	–
GWP60 MEC	5	0.1220	0.0190	0.0598	47.57	819.48	0.759	1.118	0.985
GWP60 DDI90	5	0.1418	0.0149	0.0675	59.03	705.14	0.942	0.962	1.050
GWP60 DDI98	5	0.1278	0.0159	0.0701	55.82	782.26	1.013	0.890	1.067

**Table 5:** Damaged parameters: predicted (Equations (9)–(11)) vs. estimated (Equation (1)—SPSS^®^).

	Predicted Parameters			Mean Parameter Estimates		
Sample	Equation (9)	Equation (10)	Equation (11)	Equation (1) (SPSS^®^)		
	$a\left(Y\right)$	$b\left(Y\right)$	$c\left(Y\right)$	a	b	c
	m/kN	m/kN	–	m/kN	m/kN	–
GWP55 MEC	0.0936	0.0240	0.1957	0.0936	0.0240	0.1957
GWP60 UND	0.1220	0.0190	0.0598	0.1220	0.0190	0.0598
GWP60 DDI90	0.1418	0.0149	0.0675	0.1418	0.0149	0.0675
GWP60 DDI98	0.1278	0.0159	0.0701	0.1278	0.0159	0.0701

## Data Availability

The data presented in this study are available on request from the corresponding author.
